# Supplementary material for: From sequence to enzyme mechanism using multi-label machine learning
Source: BMC Bioinformatics. 2014 May 19;15:150. doi: 10.1186/1471-2105-15-150 (PMC4229970; doi:10.1186/1471-2105-15-150)
Supplement: Additional file 2 — Java code of ml2db. Additional file ml2db_code.tar.gz contains the Java source code to run the multi-label machine learning experiments and save the results to database. The code’s Javadoc is included. [file 1471-2105-15-150-S2.zip › additional file 2/ml2db/ml2db/doc/mulan/classifier/meta/ConstrainedKMeans.html]

ConstrainedKMeans


JavaScript is disabled on your browser.


- Overview
- Package
- Class
- Use
- Tree
- Deprecated
- Index
- Help

- Prev Class
- Next Class

- Frames
- No Frames

- All Classes

- Summary:
- Nested |
- Field |
- Constr |
- Method

- Detail:
- Field |
- Constr |
- Method


mulan.classifier.meta

## Class ConstrainedKMeans

- java.lang.Object
- - weka.clusterers.AbstractClusterer
  - - weka.clusterers.RandomizableClusterer
    - - mulan.classifier.meta.ConstrainedKMeans

- All Implemented Interfaces:
  :   java.io.Serializable, java.lang.Cloneable, weka.clusterers.Clusterer, weka.clusterers.NumberOfClustersRequestable, weka.core.CapabilitiesHandler, weka.core.OptionHandler, weka.core.Randomizable, weka.core.RevisionHandler, weka.core.WeightedInstancesHandler

  ---

    

  ```
  public class ConstrainedKMeans
  extends weka.clusterers.RandomizableClusterer
  implements weka.clusterers.NumberOfClustersRequestable, weka.core.WeightedInstancesHandler
  ```

  Cluster data using the constrained k means algorithm

  Version:
  :   2012.07.16

  Author:
  :   Mark Hall, Eibe Frank, Grigorios Tsoumakas

  See Also:
  :   `RandomizableClusterer`,
      Serialized Form

- - ### Nested Class Summary

    Nested Classes

    | Modifier and Type | Class and Description |
    | --- | --- |
    | `static class` | `ConstrainedKMeans.bucketInstance` Class for representing an instance inside a bucket |
  - ### Constructor Summary

    Constructors

    Constructor and Description

    `ConstrainedKMeans()`

    the default constructor
  - ### Method Summary

    Methods

    | Modifier and Type | Method and Description |
    | --- | --- |
    | `void` | `buildClusterer(weka.core.Instances data)` Generates a clusterer. |
    | `int` | `clusterInstance(weka.core.Instance instance)` Classifies a given instance. |
    | `weka.core.Capabilities` | `getCapabilities()` Returns default capabilities of the clusterer. |
    | `weka.core.Instances` | `getClusterCentroids()` Gets the the cluster centroids |
    | `int[][][]` | `getClusterNominalCounts()` Returns for each cluster the frequency counts for the values of each nominal attribute |
    | `int[]` | `getClusterSizes()` Gets the number of instances in each cluster |
    | `weka.core.Instances` | `getClusterStandardDevs()` Gets the standard deviations of the numeric attributes in each cluster |
    | `int` | `getNumClusters()` gets the number of clusters to generate |
    | `double` | `getSquaredError()` Gets the squared error for all clusters |
    | `java.lang.String` | `globalInfo()` Returns a string describing this clusterer |
    | `java.util.Enumeration` | `listOptions()` Returns an enumeration describing the available options. |
    | `int` | `numberOfClusters()` Returns the number of clusters. |
    | `java.lang.String` | `numClustersTipText()` Returns the tip text for this property |
    | `void` | `setMaxIterations(int x)` |
    | `void` | `setNumClusters(int n)` set the number of clusters to generate |
    | `java.lang.String` | `toString()` return a string describing this clusterer |

    - ### Methods inherited from class weka.clusterers.RandomizableClusterer

      `getOptions, getSeed, seedTipText, setOptions, setSeed`
    - ### Methods inherited from class weka.clusterers.AbstractClusterer

      `distributionForInstance, forName, getRevision, makeCopies, makeCopy, runClusterer`
    - ### Methods inherited from class java.lang.Object

      `equals, getClass, hashCode, notify, notifyAll, wait, wait, wait`

- - ### Constructor Detail


    - #### ConstrainedKMeans

      ```
      public ConstrainedKMeans()
      ```

      the default constructor
  - ### Method Detail


    - #### globalInfo

      ```
      public java.lang.String globalInfo()
      ```

      Returns a string describing this clusterer

      Returns:
      :   a description of the evaluator suitable for displaying in the
          explorer/experimenter gui


    - #### getCapabilities

      ```
      public weka.core.Capabilities getCapabilities()
      ```

      Returns default capabilities of the clusterer.

      **Specified by:**
      :   `getCapabilities` in interface `weka.clusterers.Clusterer`

      **Specified by:**
      :   `getCapabilities` in interface `weka.core.CapabilitiesHandler`

      **Overrides:**
      :   `getCapabilities` in class `weka.clusterers.AbstractClusterer`

      Returns:
      :   the capabilities of this clusterer


    - #### setMaxIterations

      ```
      public void setMaxIterations(int x)
      ```

      Parameters:
      :   `x` -


    - #### buildClusterer

      ```
      public void buildClusterer(weka.core.Instances data)
                          throws java.lang.Exception
      ```

      Generates a clusterer. Has to initialize all fields of the clusterer that
      are not being set via options.

      **Specified by:**
      :   `buildClusterer` in interface `weka.clusterers.Clusterer`

      **Specified by:**
      :   `buildClusterer` in class `weka.clusterers.AbstractClusterer`

      Parameters:
      :   `data` - set of instances serving as training data

      Throws:
      :   `java.lang.Exception` - if the clusterer has not been generated successfully


    - #### clusterInstance

      ```
      public int clusterInstance(weka.core.Instance instance)
                          throws java.lang.Exception
      ```

      Classifies a given instance.

      **Specified by:**
      :   `clusterInstance` in interface `weka.clusterers.Clusterer`

      **Overrides:**
      :   `clusterInstance` in class `weka.clusterers.AbstractClusterer`

      Parameters:
      :   `instance` - the instance to be assigned to a cluster

      Returns:
      :   the number of the assigned cluster as an interger if the class is
          enumerated, otherwise the predicted value

      Throws:
      :   `java.lang.Exception` - if instance could not be classified successfully


    - #### numberOfClusters

      ```
      public int numberOfClusters()
                           throws java.lang.Exception
      ```

      Returns the number of clusters.

      **Specified by:**
      :   `numberOfClusters` in interface `weka.clusterers.Clusterer`

      **Specified by:**
      :   `numberOfClusters` in class `weka.clusterers.AbstractClusterer`

      Returns:
      :   the number of clusters generated for a training dataset.

      Throws:
      :   `java.lang.Exception` - if number of clusters could not be returned
          successfully


    - #### listOptions

      ```
      public java.util.Enumeration listOptions()
      ```

      Returns an enumeration describing the available options.

      **Specified by:**
      :   `listOptions` in interface `weka.core.OptionHandler`

      **Overrides:**
      :   `listOptions` in class `weka.clusterers.RandomizableClusterer`

      Returns:
      :   an enumeration of all the available options.


    - #### numClustersTipText

      ```
      public java.lang.String numClustersTipText()
      ```

      Returns the tip text for this property

      Returns:
      :   tip text for this property suitable for displaying in the
          explorer/experimenter gui


    - #### setNumClusters

      ```
      public void setNumClusters(int n)
                          throws java.lang.Exception
      ```

      set the number of clusters to generate

      **Specified by:**
      :   `setNumClusters` in interface `weka.clusterers.NumberOfClustersRequestable`

      Parameters:
      :   `n` - the number of clusters to generate

      Throws:
      :   `java.lang.Exception` - if number of clusters is negative


    - #### getNumClusters

      ```
      public int getNumClusters()
      ```

      gets the number of clusters to generate

      Returns:
      :   the number of clusters to generate


    - #### toString

      ```
      public java.lang.String toString()
      ```

      return a string describing this clusterer

      **Overrides:**
      :   `toString` in class `java.lang.Object`

      Returns:
      :   a description of the clusterer as a string


    - #### getClusterCentroids

      ```
      public weka.core.Instances getClusterCentroids()
      ```

      Gets the the cluster centroids

      Returns:
      :   the cluster centroids


    - #### getClusterStandardDevs

      ```
      public weka.core.Instances getClusterStandardDevs()
      ```

      Gets the standard deviations of the numeric attributes in each cluster

      Returns:
      :   the standard deviations of the numeric attributes in each cluster


    - #### getClusterNominalCounts

      ```
      public int[][][] getClusterNominalCounts()
      ```

      Returns for each cluster the frequency counts for the values of each
      nominal attribute

      Returns:
      :   the counts


    - #### getSquaredError

      ```
      public double getSquaredError()
      ```

      Gets the squared error for all clusters

      Returns:
      :   the squared error


    - #### getClusterSizes

      ```
      public int[] getClusterSizes()
      ```

      Gets the number of instances in each cluster

      Returns:
      :   The number of instances in each cluster


- Overview
- Package
- Class
- Use
- Tree
- Deprecated
- Index
- Help

- Prev Class
- Next Class

- Frames
- No Frames

- All Classes

- Summary:
- Nested |
- Field |
- Constr |
- Method

- Detail:
- Field |
- Constr |
- Method
